# Validation and Parameter Sensitivity Tests for Reconstructing Swell Field Based on an Ensemble Kalman Filter

**DOI:** 10.3390/s16122000

**Published:** 2016-11-25

**Authors:** Xuan Wang, Pierre Tandeo, Ronan Fablet, Romain Husson, Lei Guan, Ge Chen

**Affiliations:** 1 Qingdao Collaborative Innovation Center of Marine Science and Technology, College of Information Science and Engineering, Ocean University of China, Qingdao 266100, China; wangxuan_ouc@163.com (X.W.); leiguan@ouc.edu.cn (L.G.); 2 Institut Telecom/Telecom Bretagne, UMR LabSTICC, Technopôle Brest-Iroise, Brest 29280, France; pierre.tandeo@telecom-bretagne.eu (P.T.); ronan.fablet@telecom-bretagne.eu (R.F.); 3 Collecte Localisation Satellites, Brest 29280, France; rhusson@cls.fr; 4 Laboratory for Regional Oceanography and Numerical Modeling, Qingdao National Laboratory for Marine Science and Technology, Qingdao 266100, China

**Keywords:** pseudo SAR observation, EnKF, swell field reconstruction, parameter sensitivity

## Abstract

The swell propagation model built on geometric optics is known to work well when simulating radiated swells from a far located storm. Based on this simple approximation, satellites have acquired plenty of large samples on basin-traversing swells induced by fierce storms situated in mid-latitudes. How to routinely reconstruct swell fields with these irregularly sampled observations from space via known swell propagation principle requires more examination. In this study, we apply 3-h interval pseudo SAR observations in the ensemble Kalman filter (EnKF) to reconstruct a swell field in ocean basin, and compare it with buoy swell partitions and polynomial regression results. As validated against in situ measurements, EnKF works well in terms of spatial–temporal consistency in far-field swell propagation scenarios. Using this framework, we further address the influence of EnKF parameters, and perform a sensitivity analysis to evaluate estimations made under different sets of parameters. Such analysis is of key interest with respect to future multiple-source routinely recorded swell field data. Satellite-derived swell data can serve as a valuable complementary dataset to in situ or wave re-analysis datasets.

## 1. Introduction

Pervasive swells arising from prevailing westerlies propagate across ocean basins for thousands of kilometers, redistributing the specular energy transferring from fierce winds. Over the past decade, altimeters, synthetic aperture radar (SAR), and buoys have recorded this prominent signal that is transferred from the atmosphere. Swell has been investigated in the field by [1,2] to measure its ability to travel long distances, first demonstrating a low swell attenuation of 0 to 2 × 10^−7^ m^−1^. Due to overlapping waves, contamination between different wave systems is inevitable. It remains tricky to completely separate swells from wind seas to obtain a global distribution of ocean swell. Buoys can provide the directional wave spectrum to retrieve swell but they are only sparsely deployed in coastal areas and are almost nonexistent in the open ocean. Altimeter data are abundant and provide better precision for measuring wave height, but they cannot be used to discriminate between different wave systems. Taking the fully developed wind–wave relation as a separation criterion, [3] discriminated wind seas from swell climate geographical patterns using collocated altimeter/scatterometer data. SAR, currently the only instrument used globally to derive the directional spectrum, makes possible the tracking of ocean swell despite its low sample resolution. Considering frequency and angular dispersion 1/(αsinα), where α is the radian spherical distance from the source in the far field, with the aid of the SAR wave mode, the authors in [4] recomputed the dissipation rate as ranging from 3.1 to 4.0 × 10^−7^ m^−1^ for a 68% confidence level for a 400-ensemble SAR dataset from one storm case where the swell heights were perturbed independently based on the uncertainty of the SAR measurement. In another study, the range was determined to be from −0.6 to 3.7 × 10^−7^ m^−1^, as reported by [5] for 22 swell events. Recently, by recalculating the moderate wave heights captured by altimetry and SAR data, the authors in [6,7] defined swell energy dissipation as the non-breaking dissipation rate, and found this rate to range from −2.5 to 5.0 × 10^−7^ m^−1^ for 202 swell tracks and from −0.1 to 6 × 10^−7^ m^−1^ in 416 independent storms.

Satellite-observed wave datasets have been thoroughly validated and assimilated into state-of-art wave models [8,9,10,11,12] to improve the quality of wave hindcasts and forecasts. Researchers have estimated SAR swell observations for assimilation purposes, and globally distributed observations could improve wave simulation performance [13]. To survey the physical phenomena associated with swell propagation and to predict the swell heights generated by fierce storms, more realistic swell information is needed to compensate for the typically biased swell fields produced by wave models. Currently, most investigations into momentum transfer relating to wind–wave coupling at the marine boundary surface have been conducted on in situ or wave re-analysis datasets [14,15,16]. Swell datasets covering the ocean basin with real-time measurements are needed. Important as it is, there are, as yet, no sophisticated swell field data being routinely recorded. Thanks to the increasing amount of satellite data, we can now perform data assimilation, whereby sparsely distributed observed data is assimilated into physical models to create datasets with better quality. The ensemble Kalman filter (EnKF) is a state-of-the-art data assimilation technique that is applicable to both oceanic and atmospheric models [17,18,19,20], and can be used to estimate past, present and future parameter states. To improve assimilation performance, tuning the EnKF parameters, such as the ensemble size, model error, measurement error, covariance inflation, covariance localization, and balance of physical parameters, is a critical step in both simple physical models and sophisticated operational models [21,22,23,24].

Rather than a physical dynamic wave model such as WAVEWATCH 3, we use a simple predictive swell propagation model to reduce computation time, and thereafter incorporate pseudo SAR observations. From such SAR-derived pseudo swell observations, the author in [25] reconstructed swell fields using polynomial regression and the kriging interpolation scheme. Such interpolation schemes may result in poor temporal consistencies. In this respect, EnKF-based techniques [26] seem more relevant, while the reconstructed swell fields observed by SAR still require validation with buoy data to determine whether swell evolution can be represented by a simple model. In this study, we explored these two types of interpolation techniques and performed a quantitative performance analysis based on in situ buoy data. We also carried out a sensitivity analysis, which may serve as a guide for the parameterization of operational processing. The paper is organized as follows. In Section 2, we briefly introduce the datasets used in this study. The scheme of EnKF-based swell field reconstruction and storm related swell tracking in buoy data are provided in Section 3. We present a case study of a mid-latitude storm that formed on 11 January 2009 in Section 4, followed by a summary and discussion in Section 5.

## 2. Data

### 2.1. Buoy Data

We downloaded hourly buoy data processed from 20-min-long records from the National Data Buoy Center (NDBC), for which the wave height accuracy is about 0.2 m. The wave period accuracy is 1 s, and for periods greater than 10 s the resolution is larger than 1 s. The wave direction accuracy is 10° [27]. α1 and α2 are, respectively, the mean and principal wave directions, and r1 and r2 are the first and second non-dimensional normalized polar coordinates of the Fourier coefficients, respectively (http://www.ndbc.noaa.gov/measdes.shtml). We recovered the directional wave spectrum by the maximum entropy method (MEM) introduced by [28], and further partitioned it using watersheds [29] after a smoothing process [30]. Here, the wave direction is the direction from which the waves come, measured clockwise from the north.

### 2.2. SAR Data

The ENVISAT mission was launched in 2002 by the European Space Agency (ESA). With the Advanced Synthetic Aperture Radar (ASAR) instrument, wave spectra have been retrieved using quasi-linear transformation [31], and their quality has been widely reviewed [4,32]. The SAR data used here are level-2 products, further processed by [33], whose wave directional spectra are discretized every 10° in direction and whose 24 exponentially spaced wavenumbers range from 30 to 800 m. We derived the swell integral parameters from the partitioned spectra, which are the same as in the buoy data.

**Figure 1:**
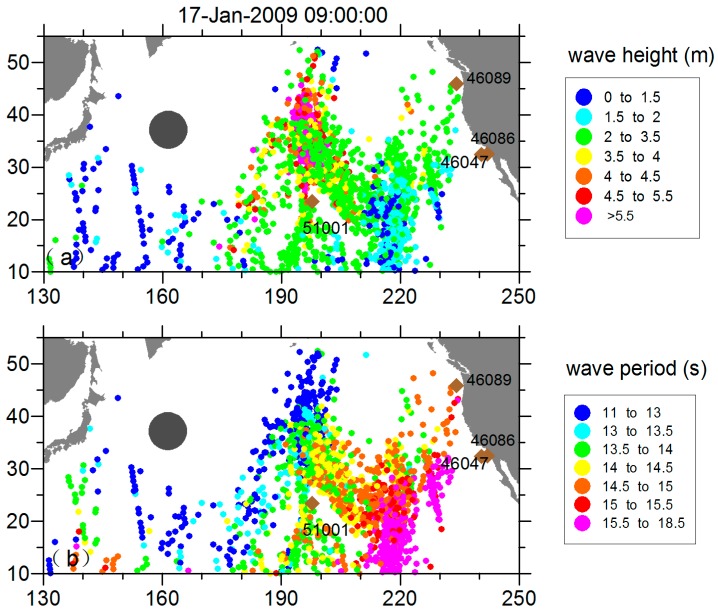
The “fireworks” of propagated swells at 17-Jan-2009 09:00:00.

The ready-made pseudo SAR data derived from the work of [25], The SAR-observed swells are artificially propagated backward by a group velocity *Cg* to identify the storm source as the spatio-temporal convergence point. When the refocused SAR observations propagate forward and backward in the ocean basin with the swell propagation model, the captured “firework” shaped swells radiating from the source (snapshots of the propagated observations) demonstrating the swell evolution are the pseudo SAR observations used in swell field reconstruction. These SAR-propagated observations that associated with a storm source are documented into one file. Figure 1 is a demonstration of “fireworks” ignited by a storm generated on 11 January 2009. The file of propagated SAR data also are archived with data quality flag and data density to guide the data control.

### 2.3. ECMWF Data

To characterize the storm, we downloaded wind and mean sea level pressure (SLP) data from the ERA-Interim global atmospheric reanalysis dataset (http://apps.ecmwf.int/datasets/data/interim_full_daily) produced by the European Centre for Medium Range Weather Forecast (ECMWF), for which data is available from 1979 to the present. The data assimilation system used to produce the ERA-Interim data is based on an updated atmospheric model, including a four-dimensional variational analysis (4D-VAR) with a 12-h analysis window.

## 3. Method

### 3.1. Ensemble Kalman Filter (EnKF)

The Kalman filter is designed to solve discrete data linear filtering problems with a Gaussian noise distribution, by minimizing the mean square error criterion. It is applicable to linear dynamic systems driven by Gaussian stochastic processes. For nonlinear systems, statistical ensemble members can be used to represent the true error covariance. This alternative statistical Monte Carlo method was termed the ensemble Kalman filter (EnKF), which offers the mean of the ensemble members evolving in the state model as a best estimate [34]. Theoretically, it asymptotically approaches the Kalman filter result if the sampling ensemble is large enough, whereas too small an ensemble number will cause covariance inaccuracy and filter divergence. The framework of EnKF with respect to swell reconstruction can be written as follows:

(1) $$x_{i}^{f}\left(t_{k}\right)=M\left(x_{i}^{a}\left(t_{k-1}\right)\right)+\eta_{i}\left(t_{k}\right)$$

(2) $$y_{i}^{f}\left(t_{k}\right)=H\left(x_{i}^{f}\left(t_{k}\right)\right)+\varepsilon_{i}\left(t_{k}\right)$$

M indicates the swell propagation model in deep water, for which the swell dissipation with a magnitude of 10^−7^ m^−1^ is omitted here and the swell energy is conveyed by the group velocity $C_{g}=\sqrt{\lambda g}/2\sqrt{2\pi }$ , which decays as 1/αsin(α) along the propagation track. Equation (1) represents the temporal dynamics of the state (i.e., swell height, wavelength, and wave direction fields) based on the physical swell propagation model with the Gaussian model error η to provide forecast fields. *H* corresponds to the spatial interpolation from the assimilation grid to the observation grid. Equation (2) includes the addition of the Gaussian measurement error ε in this spatial interpolation step. ∀i∈{1,…, N} , i represents the ensemble members. To update the estimate, the prediction with observation is weighed, as follows:

(3) $$x_{i}^{a}\left(t_{k}\right)=x_{i}^{f}\left(t_{k}\right)+K\left(t_{k}\right)d_{i}\left(t_{k}\right)$$

where *K* is the Kalman gain (associated with the priori estimation, spatial interpolation, and measurement error covariance *R*), *d* is the residual between the observation and the model forecast, and $x_{i}^{a}$ and $x_{i}^{f}$are analysis and forecast vectors, respectively. The model error matrix *Q* and measurement error matrix *R* are diagonal, which means that parameter noise is assumed to be spatially independent and identically distributed here. If the measurement error covariance approaches zero, the Kalman filter will favor the observations and Kalman gain will give more weight to the residual. Conversely, if the error covariance approaches zero, the Kalman filter will favor the model prediction. These two parameters will be given particular attention in our reported sensitivity analysis. As opposed to the 4D-VAR method, the EnKF sequentially updates the background error covariance. The flow-dependent background error covariance derived from the background estimate (ensemble forecast) determines the weight of the residual for the update. From the estimated covariance, EnKF quantifies the effect of an observation on nearby locations and the relationships between different integral wave parameter fields [35]. More details related to the EnKF scheme for swell reconstruction may be found in [26].

### 3.2. Swell Tracking from Buoy Data

As the higher frequency wave propagation slows (i.e., wave dispersion), there is a ridge-shaped frequency evolution in the in situ data, which was used by [1,2] to relocate the swell source position via the buoy wave characteristics. At a certain point, sub-indices 1 and 2 refer to wave groups of different frequencies, and *d* is the distance traveled along the great circle from the source:

(4a) $$d=C_{g1}t_{1}$$

(4b) $$d=C_{g2}t_{2}$$

**Figure 2:**
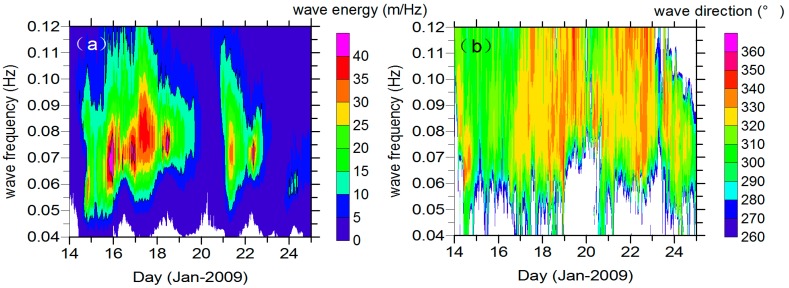
(**a**) Wave energy and (**b**) wave direction contour of buoy 51001.

**Figure 3:**
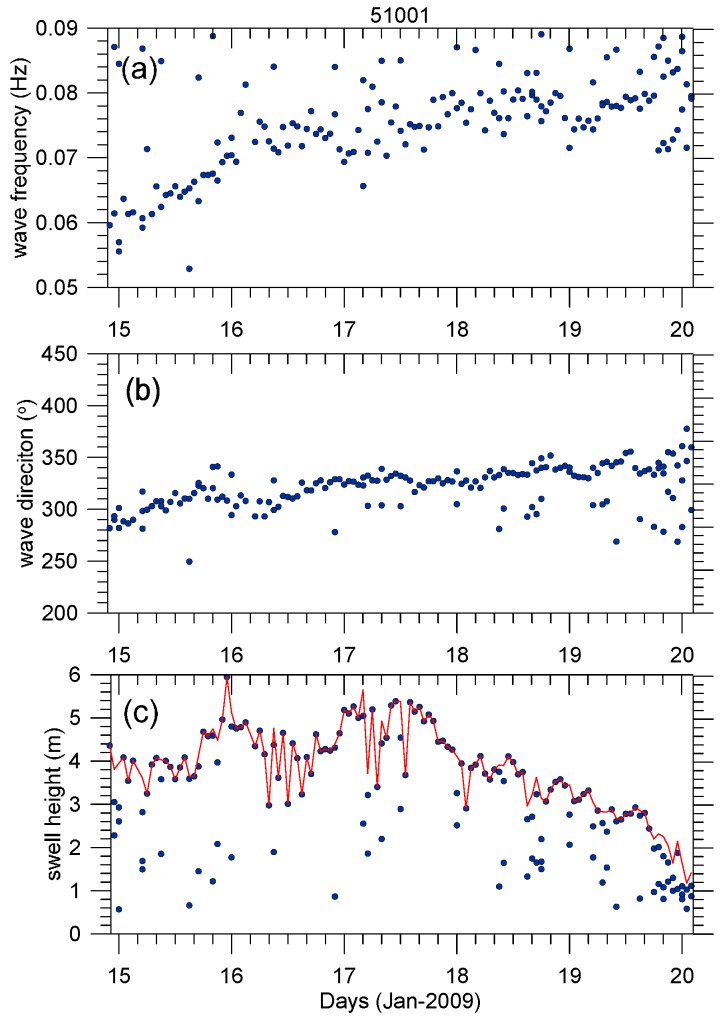
Swell partition integral parameters (**a**) swell peak frequency; (**b**) peak direction; and (**c**) significant swell height extracted from buoy 51001 overlaid with the total swell significant height. Only waves whose frequency are below 0.09 Hz are plotted.

With the group velocity given as: $C_{g}=\frac{g}{4\pi f}$, we have:

(5) $$d=\frac{g\left(t_{1}-t_{2}\right)}{4\pi \left(f_{1}-f_{2}\right)}=\frac{g}{4\pi m},$$

where m is the frequency slope relating the straight line of the wave frequency and its arrival time. As shown in Figure 2, there is a distinct wave energy ridge in the frequency signal of the 51001 buoy record from 15 to 19 January 2009, indicating a swell system coming from the northwest (at around 330° starting from the north). From 21 to 23 January 2009, another swell system is captured but at a smaller scale, lower energy, and shorter duration. We further processed the buoy data with spectrum partitions to isolate different wave systems, and calculated the integral parameters from the separated swell spectrum *R_P_*. We defined the significant swell height as $H_{ss}\;=\;4\sqrt{\iint_{R_{p}}S\left(f,\;\theta \right)dfd\theta }$, the swell peak period as the energy-weighted average period of around ±22% of the frequency of the maximum energy, and the peak direction as the energy-weighted average direction within 30° of the direction of maximum energy [25]. The partitioned swell integral parameters evolution at the buoy, as shown in Figure 3, retains the swell propagation feature. The wavelength increases, consistent with the dispersion principle, and propagates toward the southeast, and the total significant swell height slightly increases until 12:00 on Day 17 and then declines and ultimately vanishes around Day 20.

In order to retrieve swell information independently from the SAR observations, we tracked swells from the same wave systems according to the method used in [36]. A swell arriving from distant storm systems experiences large wave dispersion, but its frequency, direction, and swell height do not show much variability in the temporal neighboring points recorded by a buoy. The weighted frequency–direction–height space is $d=\sqrt{{\left(\frac{H_{s}-\bar{H_{s}}}{3\bar{H_{s}}}\right)}^{2}+{\left(\frac{\theta -\bar{\theta }}{\pi }\right)}^{2}+{\left(\frac{logf-log\bar{f}}{\frac{1}{3}}\right)}^{2}}$, which associates the swell integral parameters with the preliminary defined swell group likely to belong to the same storm source. $\bar{H_{s}}$, $\bar{\theta },$ and $\bar{f}$ represent the significant swell height, wave direction, and wave frequency average, respectively, of the previous five swell partitions of an existing wave group. If *d* is greater than 0.6, the wave integral is assigned as another wave system. Finally, all the wave groups are searched and refined to identify swell systems generated by the same storm.

## 4. Case Study

**Figure 4:**
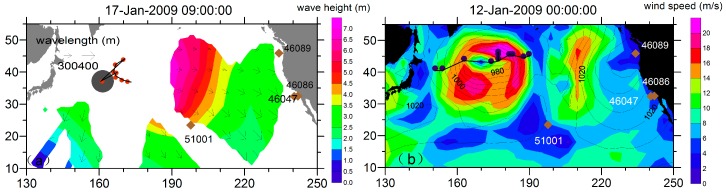
(**a**) Swell field of 17 January 2009, in which the contour represents the significant swell height, superimposed with arrows indicating the propagation direction, and the length indicating the wavelength. The gray disk indicates where the swell was generated. The red disks are the spatio-temporal refocused sources of the SAR observations with different wavelengths from 11-Jan-2009 00:00:00 to 13-Jan-2009 18:00:00 with an interval of 6 h. The diamonds indicate the locations of the buoys used to validate the synthetic swell field (buoy 32012 at 19.6° S 84.9° W not shown here); (**b**) ECMWF wind contour of 12 January 2009 overlaid with SLP to identify the origin of the storm case investigated here. The dark blue disks represent the storm movements detected by the local SLP minima from 11-Jan-2009 00:00:00 to 14-Jan-2009 00:00:00 with an interval of 6 h.

From the refocused results of the SAR wave measurements, the storm we investigated here was roughly centered at 161° E 37° N and was generated on 11 January 2009 with its radiating pseudo SAR observations. These basin-crossing observations we used sprout from 13 January 2009 all the way to 27 January 2009. The storm moved continuously toward the eastern Pacific Ocean and was finally split and perished on 17 January 2009, which tended to reinforce the right-quadrant of the trajectory, as illustrated by the ECMWF wind data. Figure 4b shows a plot of the wind speed on 12 January 2009 with the buoy positions overlapped. The directional wave spectrum provided by buoy are taken as ground truth. Table 1 shows the estimated swell frequency slope and wave direction at each buoy position range, according to the refocused swell source position (the major refocusing time was from 11-Jan-2009 00:00:00 to 13-Jan-2009 18:00:00) and the swell propagation principle. Correspondingly, Figure 4a shows the reconstructed swell field with a spatial resolution of 5° × 5°. We can observe three separate swell propagation structures that are smooth and consistent in their spatio-temporal aspect. One reason for their scattered structure is their propagation through the block of the Hawaiian Islands. Another reason is the geography of the ocean basin, in which a smaller portion of captured swell can be tracked down the western side. When these swells propagate south, they encounter the North Pacific Current. According to [37,38], waves can interact with currents and undergo refractive changes. Compared to the low swell height in the western Pacific Ocean, the eastern tendencies in swell field distribution are distinct. The swells eventually run into a “swell pool”, as first identified by [3].

Storms in the mid-latitudes always cover a vast area of 10^7^ km^2^ and span a time period of several days. To investigate the swell in the long-term scale—or rather, in the far field, the storm source is simplified as a point when establishing the swell propagation model, which we recognize to be an imperfect model representation. The swell height deviation from the propagation model is 10%–20% at 4000 km away from the ideal storm source that having a Joint North Sea Wave Project (JONSWAP) spectral shape (see Figure C1 in [4]). The SAR swell measurements are validated with the buoy data in [25]. Swells above 2 m depict an under-estimation that reaches −0.26 m. In the peak period, the most accurate results are given for values ranging between 12 s and 13.6 s, with an RMSE smaller than 0.75 s. The peak direction measurement errors exhibits no significant evolution. For the references above, Table 2 lists the EnKF parameters of the control experiment (the error here is considered to be unbiased). For wave direction, the standard deviation is added to U and V direction separately.

### 4.1. Comparison of Swell Integral Parameter Estimation

**Figure 5:**
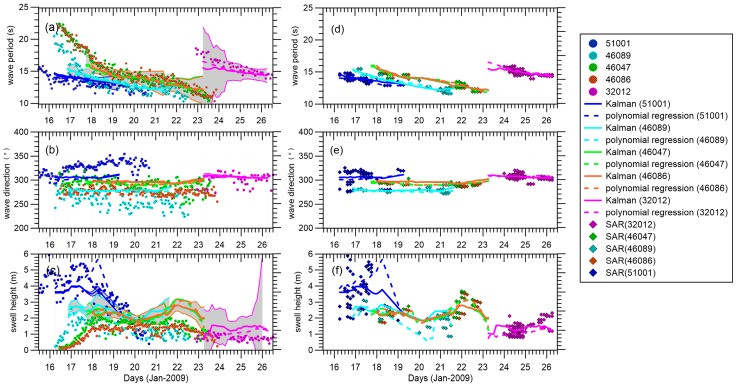
Corresponding swell events tracked at each buoy and plotted with dots with respect to (**a**) wave period; (**b**) wave direction; and (**c**) swell height. The EnKF and polynomial regression swell reconstructions are plotted with solid and dashed lines, respectively. The gray area indicates the 95% confidence interval estimated within the EnKF scheme. The (**d**–**f**) plots are the same as those of (**a**–**c**) but are superimposed with pseudo SAR data within 150 km.

Figure 5 shows a comparison of the swell integral parameters estimated by the EnKF, the polynomial-based reconstructed swell, and the buoy partitioned swell with a temporal resolution of 3 h for the reconstructed swell and 1 h for the buoy partitioned swell. Polynomial interpolation is from a global perspective and sensitive to the outliers. Also, the uneven geographical distribution of data density must be well corrected first to avoid any significant and overwhelming impact of the dense data regions on surrounding regions [25]. The results from the polynomial interpolation and EnKF are similar when the spatial distribution can be nicely represented by a spatial function with limited degrees in the wavelength and wave direction. With respect to swell height, the results for the polynomial interpolation and EnKF begin to differ. The EnKF improves the time consistency of the swell time series compared with the polynomial regression, as it takes into account past predictions. Based on the assumption of a Gaussian distribution, in Figure 5, the reconstructed fields are overlapped with a 95% confidence interval (2σ, twice the standard deviation of the ensemble spread). The ensemble spreads (i.e., the standard deviation of the wave integral parameters) are positively related to the choice of model error and increase with the assimilation time step. The frequency slopes of buoys 46086, 46047, and 46089 fit well with the estimations in Table 1, and the lowest frequencies detected in buoys 51001, 46086, 46047, and 46089 within the reconstruction time window (i.e., ignoring the forerunners whose wave energies are too low to detect in far field) show a clear wave frequency downshifting (wave period upshifting) with increased distance, as mentioned in [36]. Due to the low energy of forerunners with wave periods longer than 20 s, the wave periods captured in the SAR observations mainly range from 12 s to 17 s, as shown in Figure 5d–f, over which the available pseudo SAR observations within 150 km are superimposed.

At buoy 51001, the frequency slope of swell evolution is not as sharp as in the estimated slope and the wave direction shows a more northern trend, which could be attributed to the near-field effect and the storm approaching during the refocused time. This north-moving signal was not well represented by either the EnKF-based or polynomial-regression-based swell reconstruction methods. Buoys 46086, 46047, and 46089 are all close to the coast of the American continent and are observed by fewer SAR satellites. The reconstructed information mainly comes from the SAR observations we propagated here and the accidental inclusion of some partitioned swells generated by the approaching storm may give rise to a relatively distorted swell height. Remarkably, the buoy-detected swell heights are much lower than the reconstructed fields at buoys 46047 and 46086 during days 21–23 in Figure 5a–c. More noise can be found in buoy 46089’s partitioned integral parameters in Figure 5a–c. The maximum difference between the tracked directions by buoy 46089 can be as large as 50° and the buoy-partitioned swell heights are lower than those reconstructed from propagation of swell generated elsewhere. From the wind evolution, we find that the storm was continuously moving into the zone of buoy 46089, thus making more difficult the separation of the swell from different wave systems. Buoy 32012 seems to be a bit far away, and therefore has low wave energy. Luckily, in the boreal winter, the southern ocean is relatively calm, so this low energy may still be traceable and able to be represented by the reconstructed swell.

Also, it is interesting to examine these pseudo SAR observations at a fixed position, in addition to the reconstructed results to which they contribute. The range of the SAR retro-propagated swell directions at buoy 51001 is wider than that of other buoys, as the angular spread is stronger when closer to the storm source. The dispersive re-propagated SAR observations also indicate that the simplest swell propagation model is unlikely to work at this buoy nearest to the storm area. In the near field, the angular and dispersion spread greatly deviate from 1/αsin(α), which affects the wave energy where the reconstructed swell field does not correspond to the swell tracked by the buoy. The slight differences in wave periods of SAR observations at a fixed position may suggest a certain region of ocean’s sea state will be contributed by waves generated from a same storm but in different times or different areas within the storm, which will induce some discrepancies between the reconstructed field and the real ocean state.

**Figure 6:**
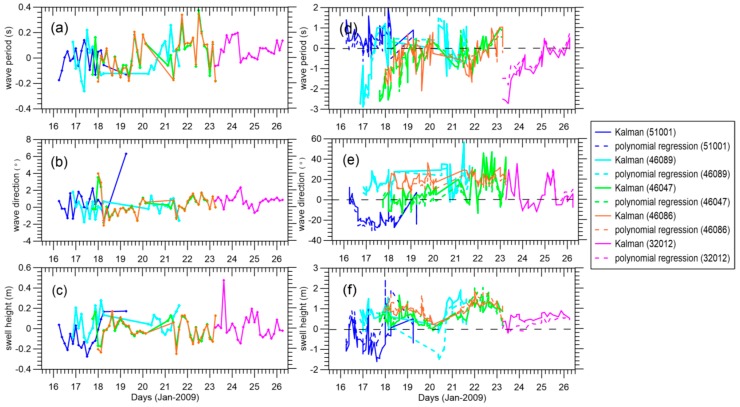
(**a**–**c**) The innovation of integral wave parameter and (**d**–**f**) the differences between the EnKF (solid line)/polynomial regression (dashed line) and buoy partitioned swells at buoys 51001, 46089, 46047, 46086, and 32012, respectively.

To explore the influence of observations in the update, Figure 6a–c show the results of the innovation of the proposed integral parameter (i.e., the difference between the analyzed and forecast ensemble means). Innovations in wave period, wave direction, and swell height range from −0.2 s to 0.4 s, −2° to 6°, and −0.2 m to 0.4 m, respectively. Except for the large increments of some integral parameters at the field edge, overall, the small oscillations in our results indicate that the model propagation and pseudo SAR observations have no conspicuous mismatches during the updating procedure. Figure 6d,f compare the differences between the three swell datasets derived from the buoy and SAR data, and show large variability as the buoy-tracked swells are not as smooth as the reconstructed ones. There are large negative wave period biases at the beginning and small positive biases at the end for buoys 46089, 46086, 46047, and 32012, which possibly indicates that the edge of the reconstructed field has lost accuracy or that the shorter and longer swells are not well-defined by the swell propagation model. The overall differences in the wave direction and swell height show a positive system bias in Figure 6d,e. Errors contributing to these differences can be two-way processes, one being the interference resulting from isolating storm-oriented swell during buoy partitioning, and another being the uncertainty of the swell propagation model when describing integral parameters in the ocean basin.

### 4.2. Parameter Sensitivity Test

**Figure 7:**
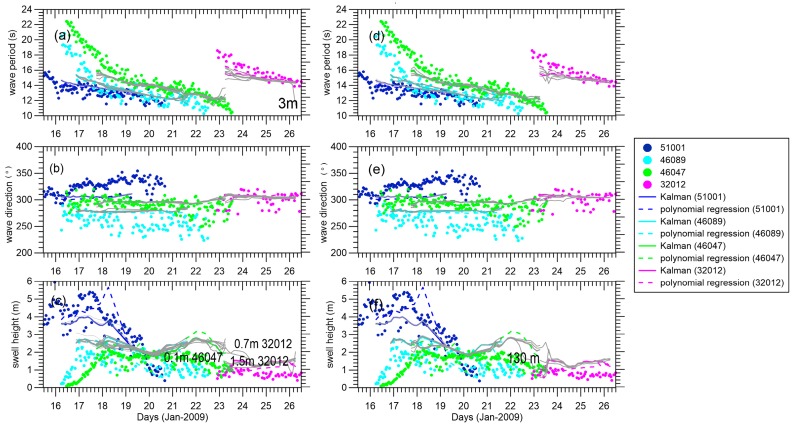
(**a**–**c**) Gray lines are EnKF results with the swell height measurement error changing as follows: 0.1, 0.3, 0.7, 1, 1.5, 2 and 3 m; (**d**–**f**) Gray lines are EnKF results with the wavelength measurement error changing as follows: 20, 40, 70, 100 and 130 m at buoys 51001, 46089, 46047, 46086, and 32012, respectively. The EnKF, polynomial regression swell reconstruction, and tracked integral wave parameters from the buoys are the same as those in Figure 5.

Tuning the EnKF parameter affected the reconstructed results. To test the sensitivity of these parameters, we ran several cases with the parameter sets listed in Table 3 and changed one parameter at a time, while the others were maintained as the control experiment. Figure 7 shows that the ensemble-mean analyses have low variability with respect to the measurement error. There is large variability only at the edge of the field. With increasing time, the divergence of the EnKF may show itself at buoy 32012 in the end. The higher we set the measurement error, more credit is given to the model. The background error is too small to incorporate the real measurements, and the EnKF is more likely to experience a divergence when propagating for a long time. In order to curb filter divergence, a prevailing method reported in the literature is the implementation of covariance inflation to increase the estimated forecast error covariance. This inflation factor is not necessarily time invariant, but requires expensive tuning to realize good performance [39].

**Figure 8:**
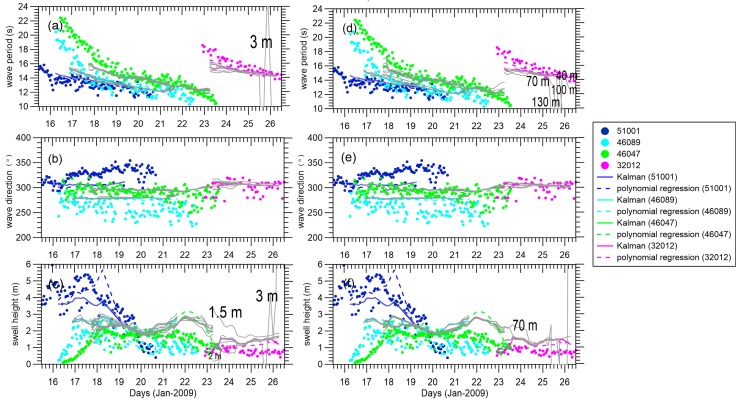
(**a**–**c**) Gray lines are EnKF results with the swell height model error changing as follows: 0.1, 0.3, 0.7, 1, 1.5, 2, and 3 m; (**d**–**f**) Gray lines are EnKF results with the wavelength model error changing as follows: 20, 40, 70, 100, and 130 m at buoys 51001, 46089, 46047, 46086, and 32012, respectively. The EnKF, polynomial regression swell reconstruction, and tracked integral wave parameters from the buoys are the same as those in Figure 5.

Figure 8 shows ensemble-mean analyses with different model errors, where the divergence lines in Figure 8a,c result from Gaussian noise with a standard deviation of 3 m in swell height added to the observation equation, Eqaution (2), and standard deviations of 40, 70, 100 and 130 m in wavelength for the divergence lines in Figure 8d,e. Increasing the model error somewhat can mitigate the divergence phenomenon. However, when the model error is set too high, ensemble spread jumps quickly and the EnKF suffers from under-sampling of the ensemble numbers and the inverse computation of the Kalman gain cannot be satisfactorily derived. If model wavelength error is increased to 70 m, the condition number of $P_{yy}+R$ is significantly increased from 23 January 2009, even if no filter divergence occurs. From 26 January 2009, the matrix is thereafter considered to be a singular matrix, while the filter divergence is very serious. To avoid divergence, the standard deviation of the model wavelength error must not exceed 40 m. Similarly, the model error for swell height should be kept below 1 m.

**Figure 9:**
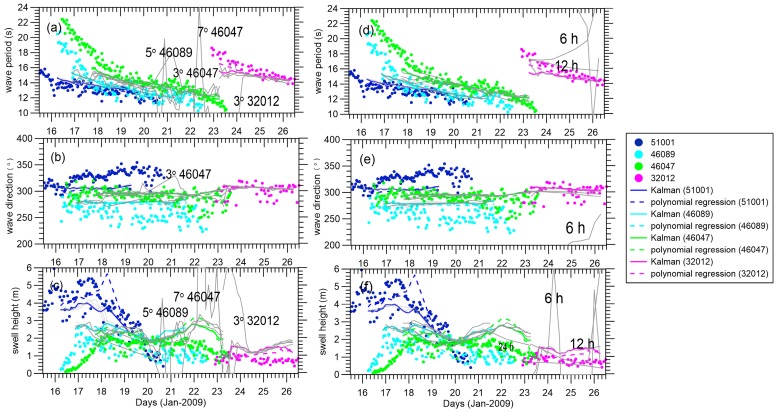
(**a**–**c**) Gray lines are EnKF results with the spatial resolution changing as follows: 3°, 5°, 7°, and 10°; (**d**–**f**) Gray lines are EnKF results with the time step changing as follows: 3, 6, 12, and 24 h. The EnKF, polynomial regression swell reconstruction, and tracked integral wave parameters from the buoy are the same as those in Figure 5.

We also tested the sensitivity to the spatial and temporal resolutions. The validations against in situ measurements in Figure 9a–c reveal the dramatic impact of spatial resolution on the accuracy of the estimated integral parameters. With finer spatial resolution, the field information is noisier and the swell field consistency seems to suffer. When finer spatial resolution is used to reconstruct the field, there is definitely an absence of real observations in some regions along the assimilation cycles. Fewer observations in the assimilation grid results in a larger ensemble spread. Increased spatial resolution leads to more under-sampling or divergence at the edge. When a larger time step is applied in the assimilation, the results become meaningless after Day 23. For time steps set to 6 h and 12 h, the wavelengths of the reconstructed field are largely distorted. These results indicate that the added error is too large for propagation over such a long distance. For a time step of 24 h, the significant swell height is lower than that of the control experiment after Day 21 at buoy 46047. A largely biased estimation of the integral wave parameters appears earlier than in cases with a shorter time step, but does not show any divergence during the assimilation process. Surprisingly, it seems the divergences are mitigated by a time step of 24 h (i.e., fewer assimilation cycles over a fixed period). Because of the added error in swell propagation, the larger time step (i.e., longer propagation distance) leads to a larger discrepancy in the predictions and observations, undermining the accuracy of the estimation and easily triggering a divergence.

The divergence in wavelength (or wave period) comes along with the divergence in swell height by changing the model error or measurement error, as the propagation speed significantly influences the distribution of the swell height field. Swells whose frequency is 0.07 Hz could travel 120 km for 3 h. If wavelength model error is set too high, the reconstructed field may not be well matched with the pseudo SAR observations and a large ensemble spread may give rise to filter divergence, like that shown in Figure 8d–f. Because propagation speed and swell height do not affect the wave direction field, wave direction seems to be the most stable of the three integral parameters, showing almost no divergence.

From the ensemble spreads of the reconstructed field, we can see where the ensemble spread is low, where the ensemble mean is not so sensitive to the parameter tuning because the observations are well-incorporated in the spatial assimilation grid and constrain the ensemble spread. The number of ensemble members is of great importance in the depiction of samples that are as close as possible to the reality. When we decreased the number of the ensemble members to 500 in our sensitivity test, the swell integral parameters did not vary much at different parameter settings, except for cases for which a large model error was applied. When the number of ensemble members was decreased to 200, due to under-sampling, the divergence appeared earlier. For the low-dimensional swell propagation model used here, it seems that 500 ensemble members are sufficient for sampling.

### 4.3. EnKF Results on Temporal Evolution Field

**Figure 10:**
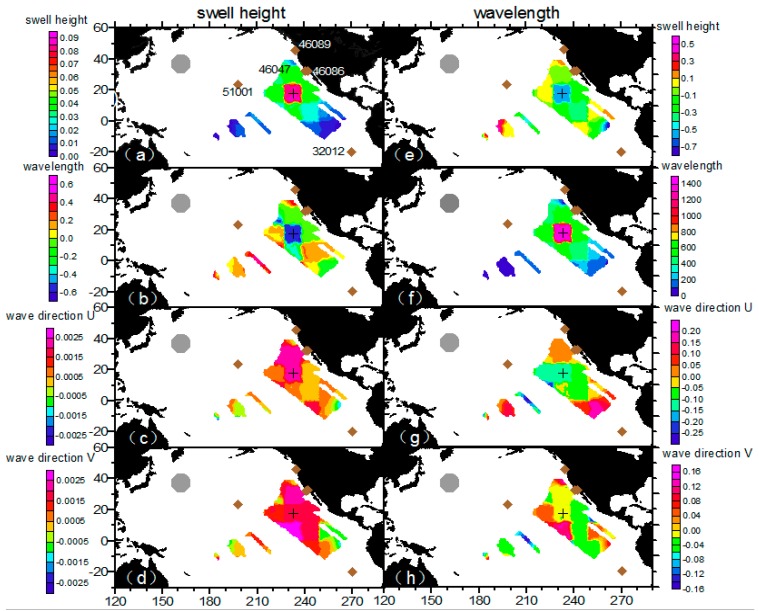
Forecast covariance of sampled state predication of swell height at observation grids and state prediction (**a**) swell height; (**b**) wavelength; (**c**,**d**) wave direction at an assimilation point (232.83° E 17.64° N) at 21-Jan-2009 09:00:00. Forecast covariance of sampled state predication of wavelength at observation grids and state prediction (**e**) swell height; (**f**) wavelength; (**g**,**h**) wave direction at an assimilation point (232.83° E 17.64° N) at 21-Jan-2009 09:00:00.

After a full examination of the EnKF results on the temporal series at fixed buoy positions, we checked the performance of the overall spatial field. In the process of computing the Kalman gain $K=P_{xy}^{f}{\left(P_{yy}^{f}+R\right)}^{-1}$, $P_{xy}^{f}=cov\left(x^{f},Hx^{f}\right)=\frac{1}{N-1}\times \overset{N}{\underset{i=1}{\sum }}\left(x_{i}^{f}\left(t_{k}\right)-{\bar{x}}^{f}\left(t_{k}\right)\right){\left(y_{i}^{f}\left(t_{k}\right)-{\bar{y}}^{f}\left(t_{k}\right)\right)}^{T}$ indicates the forecast covariance of state prediction $x^{f}$ and sampled state prediction at observation grids $y^{f}$ [40], which serves as a best estimated weight from the ensemble members to determine the contribution to be made by the observation to the assimilation grid during the updating step. However, there may be spurious correlations induced by sample noise when the number of ensemble members is limited. The impact of spurious correlations induced by sample noise can be handled using covariance localization methods, in which an optimal localization length is determined depending on the ensemble size [41]. In Figure 10, we chose a snapshot of 21-Jan-2009 09:00:00 to examine the forecast field correlation between the observation grids of three integral parameters and a fixed assimilation grid located at 232.83° E 17.64° N. Not surprisingly, the correlation between the swell height/wavelength at the fixed assimilation grid decreases with greater distance and the correlation within the spatial resolution is of similar magnitude. Wavelength and swell height show a negative correlation around the fixed point 232.83° E 17.64° N in Figure 10b,e, indicating that the results from the measured wavelength (swell height) is negatively updated at the swell height prediction (wavelength) by the correlation. Regarding the direction update, the measured swell height results shows a dipole pattern in Figure 10c,d and the measured wavelength results shows a tripole pattern in Figure 10g,h. From the computed Kalman gain, the weight of the updates is minor from the cross-correlation of the measured parameters. Basically, we can assume that the different swell parameters are independent in the assimilation.

**Figure 11:**
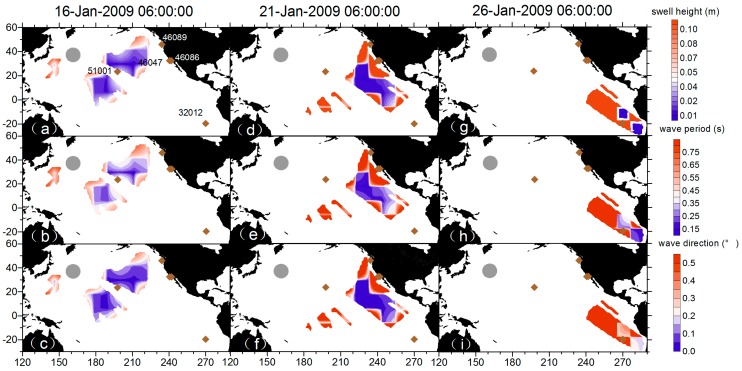
Snapshots of the spreads of swell height, period, and direction at 16-, 21-, and 26-Jan-2009 06:00:00.

**Figure 12:**
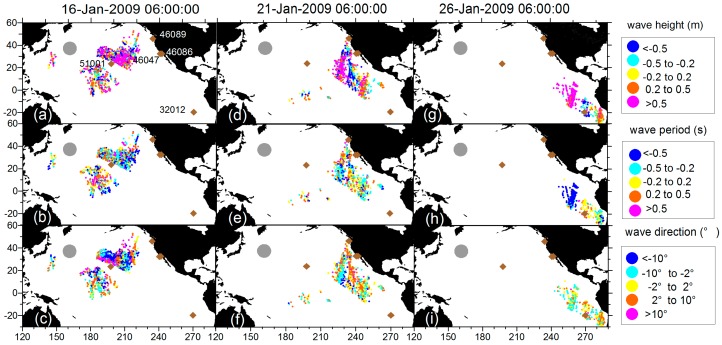
Differences in swell height, wave period, and wave direction between the EnKF field and observations at 16-, 21-, and 26-Jan-2009 06:00:00.

**Figure 13:**
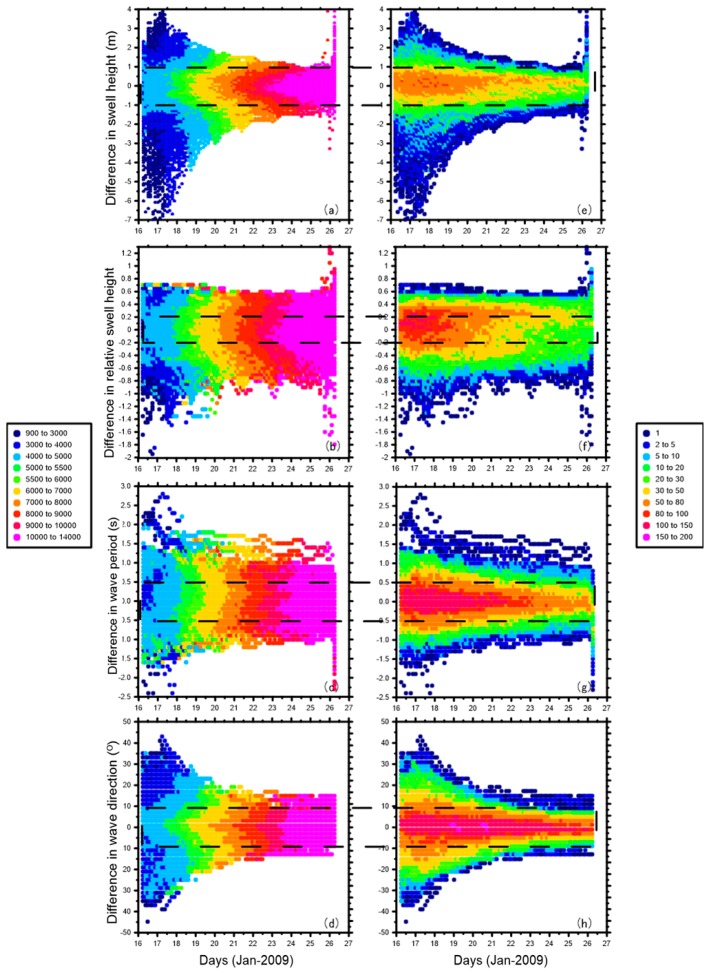
Differences in swell height (**a**,**e**); relative swell height (**b**,**f**); wave period (**c**,**g**); and wave direction (**d**,**h**) between the EnKF field and observations chronologically. Left column is overlaid with color indexes to indicate SAR observations’ mean distances from the storm, and the right column is the data density.

Figure 11 shows the temporal evolution of the ensemble spread and Figure 12 shows differences in swell height, wave period, and wave direction between the EnKF field and observations. The area that truly involves observation-driven results displays a low variance especially in the first few steps. However, along the propagation track, the lower-variance area begins to diminish. In Figure 12 at 26-Jan-2009 06:00:00, the area where the wave periods of EnKF field are smaller (slower) than the pseudo SAR observations and swell heights are higher than the pseudo SAR observations corresponds well to the large ensemble spread. It seems that the observations had insufficiently assimilated into the dynamical model, therefore the background was insufficiently corrected. During the assimilation cycles, the background estimation becomes increasingly weakly correlated to the observation grids and the influence of the observations diminishes. To illustrate how well the propagated swells have been reconstructed into an organized, smoothed field distinctly, the differences in swell height, relative swell height (divided by reconstructed swell height), wave period, and wave direction between the EnKF field and observations are plotted chronologically, overlaid with mean distances from the storm and data density (within the grid of 0.1 m × 3 h, 0.05 × 3 h, 0.1 s × 3 h, 2° × 3 h, respectively) in Figure 13. The data density clusters show that differences are mainly ±1 m, ±0.2 m, ±0.5 s, ±10° separately for swell height, relative swell height, wave period and wave direction, ensuring the good quality of the reconstructed field for other applications. Figure 13a,b and d indicate that in the sense of average, the differences tend to be smaller in long distance, and a small portion of data that located near to the storm are more likely to present large differences. This is very obvious for the swell height, as the swell decays with distance. Nevertheless, in the relative swell difference given by dividing the swell height produced by the reconstructed field, the feature still remains. In Figure 13b, the wave period shows the opposite way that in the sense of average, the differences tend to be smaller in short distance, while there is still a small portion of data that located near to the storm having large differences.

## 5. Summary and Discussion

Currently, wave models have difficulty in satisfactorily modeling swells. Reconstructed swell fields could facilitate the evaluation of pseudo SAR observations associated with storm cases to achieve better swell field simulation and to provide a new data source for further wave physics investigations. In practice, EnKF is an appealing method for assimilating satellite data (pseudo SAR observations) into dynamical models (swell propagation models) for operational use with a view to adjusting the bias that occurs in numerical models. While buoy data is always taken as ground truth in data validation, swell information cannot be directly measured like significant wave height and wind speed. Using the graphic partition procedure and tracking the swell from these partitions, we compared the swell integral parameters estimated by EnKF, polynomial-based reconstructed swells, and buoy partitioned swells for a specific storm case. Compared to polynomial regression, the EnKF improves the time consistency of the swell time series, especially with respect to swell height, and it allows for further assimilation of potential swell information from more data sources, such as buoys, altimeters, and seismic networks. From our validation, the results show that these two reconstructed swell fields both tend to underestimate the wavelength at the front edge of the field and overestimate the wavelength at the rear edge of the field. In addition, the reconstructed methods generally correspond well to the buoy partitioned swell. We consider that some of the mismatches between reconstructed and buoy partitioned swells could be due to a number of causes: SAR-derived swell or buoy partitioned swell contaminated with wave information that does not originate from the storm being investigated, the assumptions of the simplified swell propagation model, and the extra-interpolation effect at the edge of the reconstruction, etc.

Besides our validation, we also performed a sensitivity analysis with respect to the key parameters of the EnKF to provide inferences for the reconstruction of swell. Under a relatively low Gaussian model error, the reconstructed swell field based on the EnKF was rather stable and appears suitable for routinely repeated swell reconstruction. Compared to the model error, the EnkF seems to have a higher tolerance for measurement error. A decrease in spatial resolution and increase in temporal resolution seem to have a big impact on the EnKF performance and are associated with large variabilities and divergences. The swell propagation model applied to reconstruct the swell field is a low-dimensional dynamical model, so the computational cost for accurately estimating covariance by increasing the ensemble size is not expensive. We found that 500 ensemble members seems sufficient to reconstruct the swell field and 1000 ensemble members does not substantially improve the results. From the examination of field evolution, low variance occurs when there are abundant pseudo SAR observations and an enlargement of the ensemble spread at the boundary of the swell field both in magnitude and coverage over time. EnKF weighs the results by estimating the correlation between the assimilation grid and all the observation grids sampled from the ensemble. If there is any lack in observations over large areas, inaccurate estimation or divergence occurs. To mitigate this phenomenon, lower model error and coarser spatial resolution could be applied to enhance the ability to assimilate observations and reduce the increasingly large ensemble spread over time.

Obviously, the quality of the reconstructed swell field data is directly related to the pseudo SAR data. More realistic pseudo SAR data would result from a more accurate wave propagation model that considers the storm state (moving trajectory, intensity, and range of influence, etc.) and includes missing physical processes like wave interactions, the influence of local wind, and the ocean currents along the great circle. With the availability of more SAR data provided by new missions like the Sentinel-1, more high-quality swell field data will be available for demonstrating the life cycle of swell originating from fierce wind storms and propagating up to coastal areas, leading to a better understanding of wave dynamics.

## Figures and Tables

**Table 1:** Swell characteristics at each buoy position according to the swell source position.

Buoy	Distance from Source (km)	Frequency Slope (Hz/Day)	Swell Direction (°)
51001	2910–3797	0.0232–0.0177	302.1–318.5
46089	4942–5953	0.0136–0.0113	282.3–291.7
46047	6127–7056	0.0110–0.0095	295.4–303.9
46086	6239–7175	0.0108–0.0094	295.8–304.1
32012	12,026–12,948	0.0056–0.0052	302.5–310.6

**Table 2:** EnKF parameters for the control experiment.

Ensemble Members	Spatial Resolution	Time Resolution	Standard Deviation of Model Error		Standard Deviation of Observation Error	
1000	10°	3 h	swell height	0.3 m	swell height	0.3 m
			wavelength	36 m	wavelength	36 m
			wave direction (u/v direction)	0.1	wave direction (u/v direction)	0.1

**Table 3:** Parameter settings.

Ensemble Members	Spatial Resolution	Time Resolution	Standard Deviation of Model Error		Standard Deviation of Observation Error	
500	3°, 5°, 7°, 10°	3,6,12,24 h	swell height	0.1, 0.3, 0.7, 1, 1.5, 2, 3 m	swell height	0.1, 0.3, 0.7, 1, 1.5, 2, 3 m
			wavelength	20, 40, 70, 100, 130 m	wavelength	20, 40, 70, 100, 130 m
